# Leber's Hereditary Optic Neuropathy Plus Causing Recurrent Myelopathy due to an MT-DN1 Mutation at G3635A

**DOI:** 10.1155/2022/1628892

**Published:** 2022-01-11

**Authors:** Elijah Lackey, Ariel Lefland, Christopher Eckstein

**Affiliations:** Duke University Department of Neurology, Duke University Medical Center, 2905 40 Medicine Circle, Durham, NC 27710, USA

## Abstract

A 51-year-old man with known Leber's hereditary optic neuropathy (LHON) presented with worsening lower extremity weakness and numbness. Following an episode of myelopathy two years before, he had been ambulating with a walker but over two weeks became wheelchair bound. He also developed a sensory level below the T4 dermatome to light touch, pinprick, and vibration. MRI of his cervical and thoracic spine showed a nonenhancing T2 hyperintense lesion extending from C2 to T12. At his presentation two years earlier, he was found to have a longitudinally extensive myelopathy attributed to his LHON. Genetic testing revealed a 3635 guanine to adenine mutation. MRI at that presentation demonstrated a C1-T10 lesion involving the central and posterior cord but, unlike the new lesion, did not involve the ventral and lateral horns. Given the similarity to his prior presentation and a negative evaluation for alternative etiologies, he was thought to have recurrent myelopathy secondary to Leber's Plus. To our knowledge, recurrent myelopathy due specifically to the G3635A mutation in Leber's Plus has not been reported previously.

## 1. Introduction

Leber's hereditary optic neuropathy (LHON) is a maternally inherited mitochondrial disease that typically causes acute to subacute bilateral central vision loss and optic atrophy [1]. Rarely, LHON presents with systemic pathology in addition to optic neuropathy, which has been called “Leber's Plus” [1, 2]. Leber's Plus is associated with a wide variety of phenotypes, including movement disorders, psychiatric disturbances, infantile encephalopathy, skeletal disorders, and myelopathy [1, 2]. Advancements in genetic testing allow for identification of pathologic mutations causing LHON and Leber's Plus with the most common mutations, G3460A, G11778A, and T14484C, responsible for about 90 to 95% of LHON cases in the United States [3, 4]. More than 30 less common mutations have been identified, including the G3635A mutation, previously identified as pathogenic in Chinese and Russian cohorts [4–6]. Prior documented cases with the G3635A mutation experienced the acute to subacute bilateral optic neuropathy classically associated with LHON. Here, we present a patient with bilateral blindness and two distinct episodes of myelopathy secondary to Leber's Plus associated with a homoplasmic G3635A mutation. An extensive evaluation for other etiologies of myelopathy was negative. This case contributes to the understanding of the rare G3635A mutation causing Leber's Plus and describes a new clinical phenotype of bilateral subacute optic neuropathy with recurrent myelopathy.

## 2. Case Presentation

**Figure 1:**
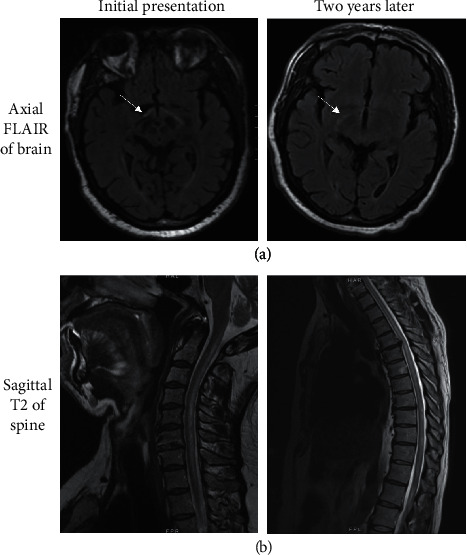
(a) MRI of the orbits showed T2 hyperintensities on FLAIR sequences to the bilateral optic nerves and optic tracts at each presentation (arrows). (b) Spinal MRIs showed longitudinally extensive nonenhancing T2 hyperintensities in distinctly different locations at each presentation.

A 50-year-old male with a history of hypertension, atrial fibrillation, and heart failure presented to a Neuroimmunology clinic with 4 months of progressive vision loss and lower extremity sensory changes. He described painless bilateral vision loss, starting with darkening of objects at distances and progressing to “dimness and darkness” of his entire visual field, including his near vision. Simultaneously, he developed bilateral lower extremity paresthesias that gradually ascended from his feet to his hips. One month prior to his Neuroimmunology clinic visit, he was admitted to an outside institution where a lumbar puncture showed mildly elevated protein of 61 mg/dL with a normal cell count of 3/*µ*L and glucose 56 mg/dL. Testing for CSF oligoclonal bands, IgG index, myelin basic protein, and serum paraneoplastic panel resulted normal (Table 1). MRI brain showed T2/FLAIR hyperintensities involving the optic nerves and tracts (Figure 1). Spinal MRI showed a C1-T10 T2 hyperintense lesion involving the central and posterior cord without contrast enhancement or edema (Figure 1). He received methylprednisolone 1 gram daily for 5 days and intravenous immunoglobulin (IVIg) 2 grams per kilogram divided over 5 days for presumed neuromyelitis optica spectrum disorder (NMOSD). His serum aquaporin-4 and myelin oligodendrocyte glycoprotein (MOG) antibodies ultimately resulted negative. His symptoms failed to improve, prompting referral to the Neuroimmunology clinic for further evaluation.

On general examination, his vital signs were normal. He was alert and oriented. Motor exam showed full strength in all extremities. Sensory exam was notable for decreased sensation to light touch and vibration in his feet to about mid-shin bilaterally. Reflexes were normal. Fundoscopic exam was notable for mild temporal optic disc pallor bilaterally. He was directly admitted for an expedited evaluation (Table 1) and additional treatment for presumed seronegative NMOSD. After plasmapheresis, he had subjective improvement in bilateral lower extremity sensation but no visual improvement. Optical computerized tomography (OCT) showed bilateral temporal retinal nerve fiber layer thinning and macular ganglion cell layer thinning consistent with focal damage to the papillomacular bundle, concerning for LHON. Humphrey visual field testing showed a nonspecific inferior defect to the right eye and left eye inferior hemifield defect that respected the midline. He was discharged home with outpatient low vision rehabilitation therapy and, due to difficulties with balance, was ambulating with a walker. Genetic testing subsequently identified a homoplasmic MT-DN1 gene mutation demonstrating a G3635A mitochondrial DNA mutation with p.Ser110Asn (S110N) variant. Given this mutation, negative workup for alternative diagnoses including the absence of antibodies for inflammatory central nervous system disorders, lack of response to empiric treatment, and an ophthalmologic exam consistent with LHON, he was diagnosed with Leber's Plus.

Approximately two years later, he presented to an emergency department with two weeks of worsening bilateral leg weakness, ascending anesthesia to his proximal legs, and bowel and bladder incontinence. He progressed from ambulating with a walker to being wheelchair bound. Physical exam demonstrated proximal greater than distal bilateral lower extremity weakness with 2/5 bilateral hip flexion, 4/5 bilateral knee flexion, 4/5 left knee flexion and extension, 3/5 right knee flexion and extension, and 3/5 bilateral ankle dorsiflexion and plantarflexion. He had a T4 sensory level to light touch, pinprick, and vibration. Reflexes were 3+ at the bilateral biceps, brachioradialis, and patellae with crossed adduction and 4+ at the Achilles with sustained clonus. Cervical and thoracic MRI showed a C2-T12 long-segment T2 hyperintense lesion. While the prior lesion involved the posterior and central spinal cord, the new lesion involved the ventral and lateral horns and extended two segments further caudally into the thoracic cord (Figure 1). Neither lesion showed contrast enhancement. Workup for inflammatory and infectious etiologies was unrevealing (Table 2). He was treated with methylprednisolone 1 gram daily for three days without significant improvement. He was discharged to a rehabilitation facility and gradually regained some of his lower extremity strength. With the occurrence of this new longitudinally extensive lesion and an unrevealing evaluation for other causes of the lesion, he was diagnosed with recurrent myelopathy secondary to Leber's Plus.

## 3. Discussion

Although 90 to 95% of cases of LHON are caused by the three most common mutations (G3460A, G11778A, and T14484C), other less common mutations are being recognized and elucidated [4, 6, 7]. To date, at least 11 primary mutations are associated with LHON, including the rare occurrence of G3635A [6]. The G3635A mutation was first described in a Russian family in 2001 and later confirmed as a primary LHON mutation in an East Asian population [5, 6]. This missense mutation at the MT-ND1 gene causes substitution of asparagine for serine, which may affect the tertiary structure of NADH dehydrogenase as part of mitochondrial complex I and cause decreased ATP synthesis during oxidative phosphorylation [6].

Patients with the G3635A mutation have previously been reported to experience acute to subacute bilateral blindness, and some had “mild neurologic symptoms” with “normal magnetic resonance imaging” [4, 5]. To our knowledge, this is the first detailed report of a patient with the G3635A mutation with bilateral blindness and MRI evidence of a recurrent longitudinally extensive myelopathy. The same case was described briefly in a prior abstract [8]. However, here we provide details regarding the case presentation, evaluation, and discussion. Besides this being the first report of recurrent myelopathy due to this mutation, another unique aspect of this case is the onset of symptoms at 50 years of age, whereas other patients with the same mutation had onset of symptoms between their teens to mid-thirties [4, 5]. An unidentified genetic, autoimmune, or metabolic influence may have resulted in this patient having late-onset symptoms with a severe myelopathy.

Potentially unidentified genetic influences in this patient include a secondary point mutation, an unidentified second mitochondrial mutation, or a substitution at another mitochondrial gene [9]. Other primary LHON mutations have variable penetrance based on secondary point mutations [9]. It has been previously suggested that a secondary point mutation, C7868T, while not a primary LHON mutation, may affect expressivity of the G3635A mutation and that individuals with both mutations are more likely to have penetrance of the disease [6]. This C7868T mutation substitutes a phenylalanine for leucine at amino acid 95 of cytochrome C oxidase subunit II [6]. It is possible that our patient has this point mutation which may account for the severity of his disease. However, it was not tested, as its presence would not change clinical management. Similarly, haplotype has been shown to affect expressivity in patients with LHON through substitutions on the MT-CYB gene [3]. Specifically, the *J* haplotype increases expressivity of the T14484C and G11778A mutations, the H haplogroup increases expressivity of G3460A, and the K haplotype reduces expressivity when paired with the G11778A mutation [3]. However, this patient's haplogroup is unknown, and there is currently no evidence for an effect of haplotype on expression of the G3635A mutation. Finally, it has been reported that two common primary LHON mutations can cause a similar phenotype with recurrent myelitis and bilateral blindness, but those mutations were not present in our patient [10, 11].

Another possibility is that there was an unidentified autoimmune trigger for his unique presentation. LHON can occur concurrently with autoimmune conditions such as multiple sclerosis (MS) and NMOSD or can mimic these autoimmune diseases by causing myelitis [12–15]. However, antibodies, including anti-MOG and anti-aquaporin-4, were consistently negative. MRI brain showed no lesions typical of MS. Additionally, there was no contrast enhancement on MRI, making an acute autoimmune inflammatory condition less likely.

Infectious and metabolic causes for myelopathy were also considered (Table 1). The patient had a low copper level (39 *µ*g/dL) after plasmapheresis but a normal level (95 *µ*g/dL) when checked again despite not receiving copper replacement. His vitamin A level was mildly elevated (101.6 *µ*g/dL). Although the patient initially screened positive for syphilis on serum VDRL, the CSF VDRL and serum RPR were both negative. Repeat serum RPR testing was negative at one and two years after his initial clinical presentation. The initial VDRL was thought to likely be a false positive. Testing for HIV and HTLV resulted negative. CSF studies including bacterial culture, fungal culture, and meningitis PCR were negative. Subsequently, there was no clear infectious or metabolic cause for his recurrent episodes of myelopathy.

This case highlights the importance of a thorough evaluation for causes of myelopathy, which may include genetic testing for diseases such as LHON. This case also serves as an important reminder that LHON can affect other parts of the nervous system beyond the optic nerves and tracts as part of Leber's Plus. Prior reports have documented myelopathy due to other primary LHON mutations, but this may be the first description of recurrent myelopathy due to the G3635A mutation with this case contributing to our understanding of the rare mutation.

Currently, the treatment for LHON and Leber's Plus is supportive. This patient received acute treatment with plasmapheresis and pooled immunoglobulins for potential concurrent autoimmune diseases such as NMO or MOG, but antibodies for these conditions were repeatedly negative. After his first presentation, he had some gradual subjective improvement to his lower extremity sensory changes but required a walker to ambulate. At his second presentation, he did not have a significant response to treatment while in the hospital and was transferred to an acute rehab facility where his lower extremity weakness gradually improved. He now requires minimal assistance for ambulation and transfers. He remains legally blind with no improvement in his vision.

## Figures and Tables

**Table 1:** Relevant cerebrospinal fluid (CSF) and serologic studies sent at initial presentation.

Test (source)	Result	Normal reference range
Leber's hereditary optic neuropathy panel (serum)	G3635A mutation with p.Ser110Asn (S110N) variant	Negative
Neuromyelitis optica spectrum IgG antibody (serum)	Negative	Negative
Myelin oligodendrocyte glycoprotein antibody (serum)	Negative	Negative
Oligoclonal bands (CSF)	None	None
IgG index (CSF)	0.48	<0.66
Myelin basic protein	<2 mcg/L	2–4 mcg/L
HIV antibody screen (serum)	Negative	Negative
HTLV-I/II antibodies (serum)	Negative	Negative
Paraneoplastic antibody panel (serum)	Negative	Negative
VDRL (serum)	Positive	Negative
VDRL (CSF)	Negative	Negative
RPR (serum)	Negative	Negative
Vitamin B12 (serum)	902 pg/ml	132–730 pg/ml
Copper (serum)	39 *µ*g/dL after first plasmapheresis but 95 *µ*g/dL on recheck	72–166 *µ*g/dL
Vitamin E (serum)	8.1 *µ*g/mL	5.0–20.0 *µ*g/mL
Vitamin A (serum)	101.6 *µ*g/dL	20–100 *µ*g/dL
Folate (serum)	32 ng/mL	>6.5 ng/mL
Heavy metal screen (serum and urine)	Negative except elevated organic arsenic which was normal on 24 hour urine collection	Negative
Meningitis PCR panel (CSF)	Negative	Negative
Bacterial and atypical mycobacterial cultures (CSF)	Negative for growth	Negative for growth

**Table 2:** Relevant cerebrospinal fluid (CSF) and serologic studies sent at second presentation, approximately two years after initial onset of symptoms.

Test (source)	Result	Normal reference range
Neuromyelitis optica spectrum IgG antibody (serum)	Negative	Negative
Myelin oligodendrocyte glycoprotein antibody (serum)	Negative	Negative
Heavy metals screen (serum)	Negative	Negative
Copper (serum)	94 ug/dL	72–166 ug/dL

## Data Availability

Data used to support the findings of this study can be obtained from the corresponding author upon request.
